# Cost-utility analysis of simultaneous initiation of finerenone and empagliflozin for type 2 diabetes mellitus complicated with chronic kidney disease

**DOI:** 10.3389/fphar.2026.1847907

**Published:** 2026-07-20

**Authors:** Qiang Liu, Jiangang Lu, Fengning Chuan, Ruobei Zhao, Na Xiong, Zheng Wang, Jian Zhang, Longbing Lai

**Affiliations:** 1 School of Business Administration, Shenyang Pharmaceutical University, Shenyang, Liaoning, China; 2 Department of Endocrinology, Chongqing Municipal Key Clinical Specialty, Chongqing University FuLing Hospital, Chongqing, China

**Keywords:** CONFIDENCE trial, cost-utility analysis, Markov model, sensitivity analysis, T2DM-CKD

## Abstract

**Background:**

Type 2 diabetes mellitus complicated with chronic kidney disease (T2DM-CKD) is a common and severe complication of T2DM that predisposes patients to end-stage renal disease (ESRD), substantially increasing mortality risk and long-term medical economic burden. Currently, SGLT-2 inhibitors are used as the foundational treatment regimen in clinical practice. As validated by the global multicenter randomized controlled CONFIDENCE trial, simultaneous initiation of finerenone plus an SGLT-2 inhibitor can further enhance renal protection and delay kidney disease progression. Despite these advancements, long-term pharmacoeconomic data supporting this combination regimen in the Chinese population remain limited.

**Objective:**

From the perspective of China’s healthcare system, this study aimed to evaluate the cost-utility of the simultaneous initiation of finerenone and empagliflozin versus empagliflozin monotherapy in T2DM-CKD patients, based on the CONFIDENCE trial design.

**Methods:**

A Markov model was constructed to simulate the natural history of T2DM-CKD. The model cycle was 1 year, with a simulation time horizon of 30 years. The willingness-to-pay (WTP) threshold was set at three times China’s 2025 *per capita* GDP (¥299,100/QALY). Base-case analysis was performed, followed by one-way and probabilistic sensitivity analyses to assess the robustness of results.

**Results:**

Base-case analysis revealed that the incremental cost-effectiveness ratio (ICER, a measure comparing the cost to the health benefit gained) of the finerenone plus empagliflozin regimen was ¥35,082.44/QALY (quality-adjusted life year), well below the WTP (willingness-to-pay) threshold. One-way sensitivity analysis indicated that the kidney disease progression coefficient (which measures the rate of worsening kidney disease) and the cost of finerenone were the most influential parameters; all parameter fluctuations within reasonable ranges retained the cost-effectiveness advantage. Probabilistic sensitivity analysis showed that the combination regimen was acceptable in 99.1% of 1,000 Monte Carlo simulations (computer-generated scenarios that model uncertainty), with all incremental QALYs positive, indicating highly robust results.

**Conclusion:**

Compared with empagliflozin monotherapy, the combined initiation of finerenone plus empagliflozin offers significant cost-utility advantages in treating T2DM-CKD. These findings provide high-quality evidence for standardized clinical care, rational pharmacotherapy, the formulation of medical insurance policies, and optimal health resource allocation in Chinese patients with T2DM-CKD.

## 1 Introduction

Chronic kidney disease (CKD) is one of the major global public health challenges threatening human health. According to the latest data from the Global Burden of Disease (GBD) Study, the total number of prevalent CKD cases among individuals aged 20 years and older worldwide reached 788 million in 2023, up from 378 million in 1990. The global age-standardized prevalence of CKD in adults was 14.2% (95% CI: 13.4%–15.2%), representing a 3.5% (95% CI: 2.7%–4.1%) increase from 1990. Meanwhile, the global age-standardized mortality rate of CKD in adults rose from 24.9 per 100,000 population in 1990 to 26.5 per 100,000 in 2023, with an increase of 6.1%. Among the top ten leading causes of death globally from 1990 to 2023, only Alzheimer’s disease and diabetes exhibited rising mortality trends, and CKD was also included in this category (GBD, 2023 Chronic Kidney Disease Collaborators, 2025). In view of this, the 78th World Health Assembly (WHA) held in May 2025 formally adopted the world’s first resolution on kidney health, listing kidney disease as a major non-communicable disease (NCD) of global priority concern (The World Health Organization, 2026).

Diabetes mellitus is one of the leading causes of chronic kidney disease (CKD) and the primary driver of kidney failure or end-stage kidney disease (ESKD). Globally, over 850 million people are affected by kidney diseases of various etiologies, among whom approximately 422 million have diabetes mellitus—this is 20 times the global cancer prevalence (42 million) or the number of people living with HIV/AIDS (36.7 million) (Al Dhaybi and Bakris, 2020). Additionally, 30%–40% of individuals with diabetes develop CKD, with diabetic patients having a 1.3–4.6-fold higher risk of CKD compared to non-diabetic counterparts. Type 2 diabetes mellitus (T2DM) constitutes the largest contributor to the burden of diabetes-related kidney disease (DKD): worldwide, incident cases of CKD attributed to T2DM increased from approximately 1.4 million in 1990 to 2.4 million in 2017, representing a 74% surge (Diabetes and Kidney Disease, 2026).

The pathophysiological mechanisms of diabetic kidney disease (DKD) are intricate. Long-term hyperglycemia-mediated metabolic disturbances and abnormal renal hemodynamics intertwine with renal inflammation and fibrotic pathways triggered by excessive mineralocorticoid receptor activation, which collectively contribute to glomerular filtration barrier injury, sustained elevation of the urinary albumin-to-creatinine ratio (UACR), and progressive renal function decline, ultimately leading to irreversible progression to end-stage renal disease (ESRD) (Tuttle et al., 2022; Alicic et al., 2018). Early clinical management centers on optimizing glycemic and blood pressure control and blocking the renin-angiotensin system; however, these strategies are unlikely to effectively halt renal inflammation and fibrosis. Moreover, single-target therapy is insufficient to disrupt the complex pathogenic network, resulting in limited disease control efficacy (Heerspink et al., 2020; The EMPA-KIDNEY Collaborative Group, 2023). In recent years, with the emergence of novel cardiorenal protective agents such as sodium-glucose cotransporter 2 inhibitors (SGLT-2i) and finerenone, a nonsteroidal mineralocorticoid receptor antagonist, clinical trials have confirmed that these two classes of agents exert complementary mechanisms of action. Their combination therapy yields synergistic effects, delaying renal function deterioration, significantly prolonging renal failure-free survival, and reducing mortality (Bakris et al., 2020; Pitt et al., 2021; Heerspink et al., 2023). Furthermore, finerenone can significantly improve UACR regardless of baseline SGLT-2i use (Rossing et al., 2022; Chen et al., 2024). At the European Renal Association (ERA) Annual Congress in June 2025, the global multicenter, double-blind, controlled CONFIDENCE trial investigating the simultaneous initiation of finerenone and SGLT-2i was prominently presented. This trial demonstrated, for the first time, that finerenone combined with empagliflozin significantly reduces UACR levels in patients with DKD, providing high-level, novel evidence for early combination therapy (Agarwal et al., 2025a). Its findings were concurrently published in the New England Journal of Medicine (NEJM), a prestigious international medical journal. A prespecified subgroup analysis of the Asian population in the CONFIDENCE trial further confirmed that simultaneous initiation of finerenone and empagliflozin significantly reduces UACR in East Asian patients with type 2 diabetes mellitus and chronic kidney disease, with a favorable safety profile (Agarwal et al., 2025b). Given the remarkable clinical efficacy of simultaneous initiation combination therapy, it is essential to further explore its economic value.

The increasing prevalence, low awareness, and economic burden of CKD and DKD in China underscore the urgent need for improved intervention strategies. Given the high costs and poor prognosis associated with late-stage disease and current treatment patterns, evaluating new cost-effective therapies is crucial. Therefore, this study will analyze the cost-utility of simultaneously initiating finerenone combined with empagliflozin from the perspective of China’s healthcare system, aiming to inform clinical practice, resource allocation, and insurance policy development.

## 2 Materials and methods

### 2.1 Target population

The target population of this study was consistent with the Asian prespecified subgroup enrolled in the CONFIDENCE trial.

Inclusion criteria were: age ≥18 years; confirmed type 2 diabetes mellitus; stage 2–4 chronic kidney disease (eGFR 15–89 mL min^-1^·[1.73 m^2^]^−1^); urinary albumin-to-creatinine ratio 100–5,000 mg/g; treatment with maximum tolerated dose of ACEI or ARB for at least 1 month.

Exclusion criteria were: maintenance hemodialysis or peritoneal dialysis; post-renal transplantation; severe hepatic insufficiency; other severe chronic diseases such as malignancies.

### 2.2 Model structure

Based on existing literature, a 6-state Markov model was constructed using Microsoft Excel. The model included the following health states: normoalbuminuria (NA), microalbuminuria (MAU), macroalbuminuria (MA), hemodialysis (HD), renal transplantation (RT), and death (absorbing state). The transition directions among health states were set in accordance with the natural history of type 2 diabetes mellitus with chronic kidney disease (T2DM-CKD): bidirectional progression or regression was allowed among normoalbuminuria, microalbuminuria, and macroalbuminuria; macroalbuminuria could progress to hemodialysis; transitions between hemodialysis and renal transplantation were permitted; and all non-death states could transition to death, which was defined as an absorbing state with no outward transitions.

**FIGURE 1:**
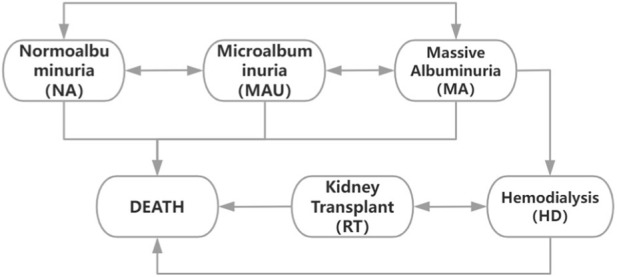
Markov model diagram.

Although patients enrolled in the CONFIDENCE trial had baseline UACR levels of 100–5,000 mg/g (i.e., MAU or MA states), the combination therapy achieved a 52% reduction in UACR from baseline at 180 days, with reductions reaching 62% in moderate-to-low risk patients. Thus, some patients are expected to achieve normalization of UACR (<30 mg/g) during long-term treatment. Therefore, the NA state was incorporated into the model with bidirectional transitions between MAU and NA to capture the potential for complete reversal of albuminuria—the optimal clinical benefit of combination therapy. This design not only allows the model to more comprehensively reflect the potential benefits of combination therapy but also enables sensitivity analyses to thoroughly examine how uncertainty in this transition pathway affects the conclusions, as illustrated in Figure 1.

Given that hyperkalemia events in the combination therapy group were observed to be infrequent, predominantly mild-to-moderate, and clinically manageable in the CONFIDENCE trial, with no significant impact on treatment adherence or long-term disease progression; and considering that other common adverse reactions of the study medications (empagliflozin and finerenone) were mostly mild-to-moderate, transient, of low incidence, and without long-term disease impact, with their associated costs and utility variations already incorporated into the management costs of the corresponding disease states, we—in line with the principle of model parsimony in pharmacoeconomics—did not establish a separate health state for adverse reactions, including hyperkalemia, which would not affect the core conclusions of this study (Shiau et al., 2024).

Given the remaining life expectancy of enrolled patients, the disease progression patterns of type 2 diabetes mellitus with diabetic kidney disease (T2DM-DKD), and the clinical application characteristics of relevant treatments, this study set the model cycle to 1 year and the total simulation time horizon to 30 years. In addition, because changes in patients’ health states may occur at any time within a single cycle, the model implemented half-cycle correction to more accurately simulate patient state transitions.

### 2.3 Markov model parameters

In this study, the Markov model incorporated costs, utility values, and transition probabilities between health states. As this analysis was conducted from the perspective of the healthcare system and adverse reactions were simplified in the model setting, only direct medical costs were included, comprising drug costs, annual management costs for each disease state, and specialized costs for end-stage renal disease (ESRD).

The drug dosages were determined in accordance with the CONFIDENCE trial protocol and the manufacturers’ package inserts: empagliflozin was administered at 10 mg once daily (qd), with a unit price of ¥4.24 per tablet (10 mg); finerenone was administered at its long-term target maintenance dose of 20 mg qd, with a unit price of ¥6.45 per tablet (10 mg). Annual drug costs were calculated based on 365 days of treatment per year, as detailed in Table 1.

**TABLE 1:** Cost parameters.

Parameter	Value	Range	Source
Examination Costs	242.64	194.11–291.17	Yang et al. (2025), Wei et al. (2023)
Annual Management Cost for Microalbuminuria (MAU) State (CNY/year)	203.41	162.73–244.1	Yang et al. (2025), Wei et al. (2023)
Annual Management Cost for Macroalbuminuria (MA) State (CNY/year)	5,730.15	4,584.12–6,876.18	Yang et al. (2025), Wei et al. (2023)
Annual Cost of Empagliflozin 10 mg (CNY/year)	1,547.6	1,238.08–1857.12	NHSA
Annual Cost of Finerenone 20 mg (CNY/year)	4,708.5	3,766.8–5,650.2	NHSA
Annual Cost of Background Pharmacotherapy (CNY/year)	3,567.55	2,854.04–4,281.06	Yang et al. (2025)
Annual Cost of Hemodialysis (HD) (CNY/year)	150,379.52	120,303.61–180455.42	Yang et al. (2025), Hong et al. (2023)
First-Year Cost of Renal Transplantation (RT) (CNY)	182,791.88	146,233.50–219350.25	Yang et al. (2025), Hong et al. (2023)
Annual Cost of Renal Transplantation (RT) in Subsequent Years (CNY)	145,587.07	116,469.65–174704.48	Yang et al. (2025), Hong et al. (2023)

In this study, the empagliflozin monotherapy group was designated as the control group, and baseline transition probabilities between health states were derived from the relevant literature, based on Chinese population-based studies. For the combination therapy group, parameter calibration of transition probabilities between health states was performed using data from the control group. This study adopted the Progression Coefficient (PC) to calibrate the transition probabilities in the combination therapy group, thereby quantifying the delaying effect of the combined intervention on the progression of diabetic kidney disease (DKD).

Change in urinary albumin-to-creatinine ratio (UACR) has been widely accepted as a valid surrogate endpoint for evaluating kidney disease progression in chronic kidney disease (CKD) clinical trials. At a scientific workshop jointly organized by the National Kidney Foundation (NKF), the U.S. Food and Drug Administration (FDA), and the European Medicines Agency (EMA) on 16 March 2018, early change in UACR was formally recognized as a candidate surrogate endpoint for CKD clinical trials (Levey et al., 2020). Heerspink et al. (2019) established, through a meta-analysis, the quantitative relationship between UACR change and CKD clinical endpoints, demonstrating that every 30% reduction in UACR corresponds to a hazard ratio of 0.68 (95% Bayesian credible interval: 0.47–0.95) for CKD progression, which formed the direct basis for the quantitative derivation of the Progression Coefficient (PC) in this study. Building on this, Heerspink et al. conducted further research and published an individual participant data meta-analysis (IPD-MA) in 2026, incorporating 48 randomized controlled trials with a total of 85,681 participants, which further confirmed that every 30% UACR reduction was associated with a 19% lower risk of kidney failure or doubling of serum creatinine, with an overall coefficient of determination (R^2^) of 0.66 (95% BCI 0.06–0.98) (Heerspink et al., 2026). Notably, in the diabetes subgroup (18 trials, 78,096 participants), the R^2^ reached 0.72, suggesting that UACR as a surrogate endpoint has stronger predictive validity in the T2DM-CKD population (Goulooze et al., 2022). Furthermore, the dose–exposure–response analysis from the FIDELIO-DKD trial demonstrated that the first-year reduction in UACR achieved with finerenone significantly predicted its subsequent long-term effect in slowing eGFR decline (Tuttle et al., 2022). The CONFIDENCE trial adopted UACR as its primary endpoint, given its established role as a practical surrogate for CKD progression (Agarwal et al., 2025a; Agarwal et al., 2025b). Therefore, this study calibrated long-term disease progression probabilities based on short-term UACR changes.

Based on the above theoretical rationale, this study further quantitatively calculated the PC using the results from the Asian subgroup analysis of the CONFIDENCE trial. The relevant clinical evidence and calculation procedures are detailed below.

#### 2.3.1 Additional UACR reduction

Based on the results of the prespecified Asian subgroup analysis of the CONFIDENCE trial, empagliflozin combined with finerenone achieved an additional 34% reduction in UACR compared with empagliflozin monotherapy (Agarwal et al., 2025b).

#### 2.3.2 Quantitative relationship between UACR reduction and kidney disease progression risk

Based on published data from relevant studies, for every 30% reduction in UACR, the hazard ratio (HR) for kidney disease progression in patients with type 2 diabetes mellitus was 0.68 (Heerspink et al., 2019).

#### 2.3.3 Progression coefficient (PC) for kidney disease progression

Based on the log-linear relationship between UACR reduction and the risk of kidney disease progression, the calculation formula for the Progression Coefficient (PC) in the combination therapy group (i.e., the ratio of progression probabilities in the combination group relative to the monotherapy group) is as follows:

$$PC={HR}^{\Delta UACR/30\%}$$

When the relevant parameters were substituted into the formula, a kidney disease Progression Coefficient (PC) of 0.65 was obtained. Since the log-linear relationship between UACR reduction and CKD progression risk was primarily validated for early albuminuria-related outcomes rather than for ESRD hard endpoints such as hemodialysis; moreover, the pathophysiological mechanisms driving the transition from macroalbuminuria to hemodialysis are more complex and involve progressive eGFR decline, which cannot be fully captured by UACR changes alone; and long-term data from the FIDELIO-DKD trial also suggest that the effect of finerenone on ESRD endpoints may be partially independent of its UACR-lowering effect. Therefore,in this study, only transitions associated with CKD progression were calibrated; all other transition pathways, which are unrelated to the pharmacological mechanism of the combination therapy, retained the baseline parameters from the control group. The relevant transition probability data and their sources for the control group and combination therapy group are presented in Table 2.

**TABLE 2:** Transition probability among different states.

Group	Transition pathway	Transition probability	Range	Source
Empagliflozin Monotherapy Group	NA→MAU	0.059	0.053–0.065	Yang et al. (2025), Li et al. (2021), Cherney et al. (2017), Mayer et al. (2019)
	NA→MA	0.011	0.010–0.013	Yang et al. (2025), Li et al. (2021), Cherney et al. (2017), Mayer et al. (2019)
	MAU→MA	0.008	0.007–0.009	Yang et al. (2025), Li et al. (2021), Cherney et al. (2017), Mayer et al. (2019)
	MAU→NA	0.134	0.121–0.147	Yang et al. (2025), Mayer et al. (2019)
	MA→MAU	0.134	0.121–0.147	Yang et al. (2025), Mayer et al. (2019)
	MA→NA	0.015	0.014–0.017	Yang et al. (2025), Mayer et al. (2019)
Combination Therapy Group	NA→MAU	0.038	0.034–0.042	Progression Coefficient
	NA→MA	0.007	0.007–0.008	Progression Coefficient
	MAU→MA	0.005	0.004–0.005	Progression Coefficient
	MAU→NA	0.134	0.121–0.147	Progression Coefficient
	MA→MAU	0.134	0.121–0.147	Progression Coefficient
	MA→NA	0.015	0.014–0.017	Wu et al. (2018)
Common Transition Probability	MA→HD	0.088	0.079–0.097	Wu et al. (2018)
	HD→RT	0.006	0.006–0.007	Yang et al. (2021)
	RT→HD	0.035	0.032–0.039	Tong et al. (2007)
	NA→DEATH	0.005	0.005–0.006	Tong et al. (2007)
	MAU→DEATH	0.008	0.007–0.008	Tong et al. (2007)
	MA→DEATH	0.009	0.008–0.009	Tong et al. (2007)
	RT→DEATH	0.037	0.033–0.041	Yang et al. (2021)
	HD→DEATH	0.042	0.038–0.046	Yang et al. (2021)

In this study, quality-adjusted life years (QALYs) were used as the measure of health outcomes, calculated as the survival time of patients in a given health state multiplied by the corresponding health utility value. Utility values were derived from previously published relevant studies, incorporating data from studies in other countries and extrapolations from observational research conducted in the Chinese context, as detailed in Table 3. It should be emphasized that the utility value of 0.92 for the MAU state was based on the following considerations: (1) as an early pathological stage of DKD, MAU should carry a health utility lower than NA but higher than MA; (2) referring to the study by Zhang et al. (2023), utility values in CKD patients decreased progressively with increasing disease severity, with a decrement of 0.022 from CKD stage 1 to stage 2. The MAU utility of 0.92 represents a reduction of 0.016 from the NA utility of 0.936, which is smaller than the aforementioned decrement. This conservative estimate was further validated in subsequent sensitivity analyses.

**TABLE 3:** Utility parameters.

Group	Transition pathway	Transition probability	Range
NA	0.936	0.842–0.992	Yang et al. (2025), Li et al. (2021)
MAU	0.920	0.828–0.966	Yang et al. (2025), Li et al. (2021), Zhang et al. (2023)
MA	0.906	0.815–0.960	Yang et al. (2025), Li et al. (2021), Zhang et al. (2023)
HD	0.798	0.758–0.838	Yang et al. (2025), Zhang et al. (2023)
RT	0.730	0.657–0.774	Yang et al. (2025), Yang et al. (2021)

The incremental cost-utility ratio (ICUR) was adopted as the indicator to quantify the difference in cost per unit of utility between the control and combination therapy groups. In accordance with the recommendations of the Chinese Guidelines for Pharmacoeconomic Evaluations (2020 Edition), a willingness-to-pay (WTP) threshold of 3 times China’s *per capita* GDP in 2025 (99,700 yuan, or 299,100 yuan) was applied. A 5% discount rate was set for both costs and health outcomes.

### 2.4 Sensitivity analyses

In accordance with the recommendations of the Chinese Guidelines for Pharmacoeconomic Evaluations (2020 Edition), one-way sensitivity analysis and probabilistic sensitivity analysis (PSA) were performed to verify and assess the model’s robustness and the stability of the results.

For a one-way sensitivity analysis, the input parameters were varied within their prespecified ranges (±20%) to examine the magnitude of impact of individual parameter changes on the model output (i.e., ICUR), and a corresponding tornado plot was generated.

PSA was conducted using Monte Carlo simulation (MCS). Appropriate prior distributions were assigned to all key parameters based on their distributional characteristics: cost parameters followed a Gamma distribution, probability parameters followed a Beta distribution, and utility parameters followed a Normal distribution. A total of 1,000 iterative simulations were carried out. Meanwhile, an incremental cost? The effectiveness scatter plot and the cost? The effectiveness-acceptability curve (CEAC) was plotted (Huang et al., 2025a).

To thoroughly evaluate the potential uncertainty inherent in extrapolating short-term UACR data to 30-year long-term clinical benefits, sensitivity analyses were specifically performed on the Progression Coefficient (PC). In one-way sensitivity analysis, the PC was varied over a ±20% range around its base-case value of 0.65 (i.e., 0.52–0.78). This range broadly covers plausible alternative hazard ratio (HR) assumptions for the association between UACR reduction and the risk of CKD progression, and is equivalent to systematically testing scenarios in which each 30% reduction in UACR corresponds to an HR between 0.55 and 0.82. In probabilistic sensitivity analysis, the PC was modeled with a Beta distribution to capture its inherent stochastic uncertainty as a probability parameter, thereby comprehensively evaluating the potential impact of random fluctuations in this parameter on the base-case conclusions over the 30-year simulation horizon.

### 2.5 Analysis software

In accordance with the recommendations of the Chinese Guidelines for Pharmacoeconomic Evaluations (2020 Edition), all the aforementioned modeling and analytical procedures were performed using Microsoft Excel 2019.

## 3 Results

### 3.1 Base-case results

Base-case results are in Table 4. Over 30 years, starting empagliflozin and finerenone together provided greater health benefits than empagliflozin alone, with an additional 0.20 QALYs. The combination raised total costs by ¥7,103.23. The ICUR was ¥35,082.44/QALY, well below the willingness-to-pay (WTP) threshold in this study (Chinese Pharmaceutical Association, 2020). Thus, simultaneous initiation of empagliflozin and finerenone is more cost-effective than empagliflozin alone.

**TABLE 4:** The results of base-case results.

Indicator	Control group	Combination therapy group	Increment
Cost (¥)	263,778.60	270,881.83	7,103.23
Utility (QAIY)	13.02	13.22	0.20
ICER(¥/QALY)	—	—	35,082.44

### 3.2 One-way sensitivity analysis results

**FIGURE 2:**
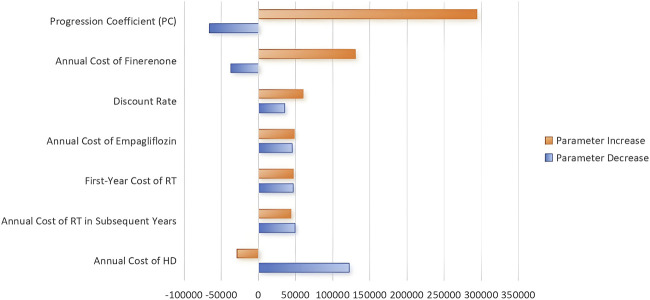
One-way sensitivity analysis results.

To evaluate the impact of parameter variations on model outcomes, one-way sensitivity analysis was performed on key input parameters, and a tornado diagram was generated to visually illustrate the marginal effects of each parameter on the incremental cost-utility ratio (ICER) within predefined ranges (Figure 2). In Figure 2, the horizontal axis represents the variation in ICER, while the vertical axis ranks parameters in descending order of influence on ICER. Red and blue bars represent ICER values when parameters were set to their upper and lower bounds, respectively.

The results demonstrated that the Progression Coefficient (PC) was the most influential parameter. Across the PC range of 0.52–0.78, the ICER consistently remained below the willingness-to-pay (WTP) threshold of ¥299,100/QALY, indicating that the long-term cost-utility advantage of the combination therapy remained robust under both conservative and optimistic surrogate-endpoint assumptions.

The costs of finerenone, the discount rate, the cost of empagliflozin, the first- and subsequent-year costs of renal transplantation, and annual dialysis costs had relatively limited effects on the ICER. Within the predefined ranges of all parameters, the ICER remained below the WTP threshold, with no scenario substantially altering the base-case conclusions. These findings suggest that, even accounting for the inherent uncertainty of extrapolating short-term UACR data to 30-year long-term clinical benefits, the base-case results remain highly robust and credible (Huang et al., 2025b).

### 3.3 Probabilistic sensitivity analysis results

**FIGURE 3:**
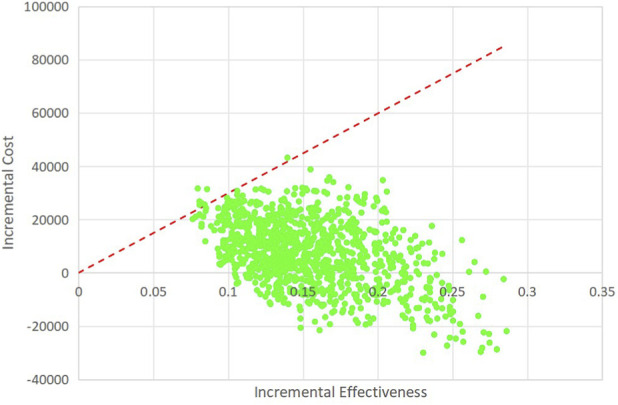
Incremental cost-utility scatter plot.

**FIGURE 4:**
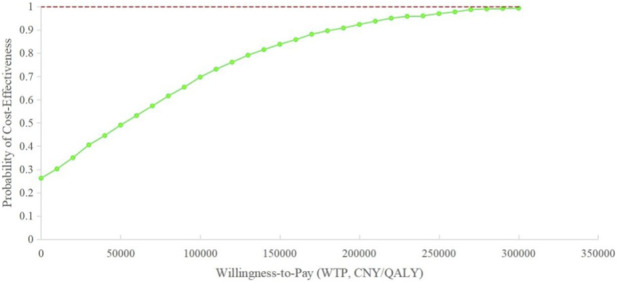
Cost-effectiveness acceptability curve (CEAC).

To comprehensively evaluate the impact of model parameter uncertainty on the study’s conclusions, a probabilistic sensitivity analysis (PSA) was conducted. A Monte Carlo simulation was used to conduct 1,000 iterations for all key model parameters (including drug costs, dialysis costs, kidney disease progression coefficient, discount rate, etc.). An incremental cost-utility scatter plot (Figure 3) and a cost-utility acceptability curve (CEAC, Figure 4) were simultaneously generated to quantify the impact of parameter fluctuations on the cost-effectiveness of the combination therapy regimen.

#### 3.3.1 Incremental cost-utility scatter plot

In Figure 3, the horizontal axis represents the incremental quality-adjusted life years (ΔQALY) of the combination therapy relative to monotherapy, denoting the incremental health benefit; the vertical axis represents the incremental cost (ΔC) of the combination therapy relative to monotherapy, denoting the incremental economic burden. The red dashed line indicates the willingness-to-pay (WTP) threshold, with the area below it representing the cost-utility acceptable region and the area above it the unacceptable region. The results shown in Figure 3 are as follows:

1. All 1,000 simulated scatter points are entirely distributed to the right of the 0 mark on the horizontal axis, meaning all ΔQALY values are positive. This demonstrates that no efficacy reversal occurred in any simulation, confirming that the combination therapy consistently delivers significantly greater health benefits than monotherapy across the full range of random parameter fluctuations. This finding is highly consistent with the pharmacological mechanism of finerenone combined with empagliflozin in delaying the progression of diabetic kidney disease (DKD), fully verifying the clinical rationality of the model.
2. Most scatter points are located in the first quadrant (ΔC > 0), indicating that the combination therapy achieves additional health benefits with a certain increase in cost, which aligns with the cost characteristics of combination pharmacotherapy. Meanwhile, a portion of the scatter points is located in the fourth quadrant (ΔC < 0), indicating that the combination therapy has the potential to achieve both health benefits and cost savings simultaneously, thereby presenting an absolute cost-utility advantage.

Notably, across the 1,000 Monte Carlo simulations, despite the Progression Coefficient (PC) randomly fluctuating within its predefined Beta distribution, all simulated ICER values remained consistently below the WTP threshold. This further confirms, at a probabilistic level, that the economic advantage of the combination therapy remains robust even after fully accounting for the stochastic uncertainty associated with extrapolating PC as a surrogate endpoint over the 30-year horizon.

3. Almost all scatter points lie below the WTP line, with only a very small number of points distributed above the line. This indicates that, in the vast majority of parameter-fluctuation scenarios, the incremental cost-utility ratio (ICUR) of the combination therapy is below the statutory WTP threshold, and the combination therapy has a clear cost-utility advantage.

#### 3.3.2 Cost-effectiveness acceptability curve

In Figure 4, the horizontal axis represents different values of the WTP threshold, and the vertical axis represents the probability that the combination therapy regimen is cost-effective. The results show that the acceptability probability of the combination therapy increases rapidly as the WTP threshold rises. At the statutory WTP threshold of ¥299,100/QALY, the acceptability probability of the combination therapy reaches 99.1%, which is fully consistent with the conclusion that the vast majority of points in the scatter plot lie below the WTP line, further verifying the robustness of the model results.

## 4 Discussion

### 4.1 Main findings

The intervention regimen in this study was strictly designed based on the CONFIDENCE clinical trial—the world’s first multicenter, double-blind, double-dummy randomized controlled trial (RCT) investigating the simultaneous initiation of finerenone and empagliflozin in patients with type 2 diabetes mellitus and chronic kidney disease (T2DM-CKD). Its results were published in the New England Journal of Medicine in 2025. A 1-year Markov cycle model with a 30-year continuous simulation horizon was constructed to mimic the long-term disease course of T2DM-CKD patients and systematically evaluate the cost-utility of finerenone plus empagliflozin versus empagliflozin monotherapy.

Results showed that combination therapy significantly improved health outcomes compared with monotherapy, with an incremental cost-effectiveness ratio (ICER) of ¥35,082.44/QALY—far below China’s pharmacoeconomic threshold of three times the *per capita* GDP (¥299,100/QALY). The robustness of the model and the findings was fully verified through one-way and probabilistic sensitivity analyses. These results indicate that the combination strategy based on the CONFIDENCE protocol holds a distinct cost-utility advantage in the Chinese T2DM-CKD population.

### 4.2 Comparison with existing literature

Most domestic and international pharmacoeconomic studies on T2DM-CKD have focused on SGLT-2 inhibitor monotherapy or sequential treatment, whereas long-term economic evidence for the simultaneous initiation of finerenone and SGLT-2i remains scarce (Mosenzon et al., 2021; Kluger et al., 2018). The CONFIDENCE trial and its Asian subgroup first demonstrated that simultaneous combination therapy led to a significantly greater reduction in UACR from baseline than either monotherapy. With Asian patients accounting for 46% of the trial population, the study provided direct evidence-based support for research in Chinese populations.

This study derived the kidney disease progression coefficient from the Asian subgroup effects in this trial and used China-specific medical cost data, thereby filling a gap in the long-term economic evaluation of this combination regimen in China. Compared with similar modeling studies in Europe and the United States, the present study better aligns with China’s disease profiles and health economic system, rendering its results more generalizable.

### 4.3 Implications for healthcare policy

In China, one to three times the *per capita* GDP is currently used as the reference WTP threshold for pharmacoeconomic evaluations. This study adopted three times the *per capita* GDP (¥299,100/QALY) as the decision criterion, and the ICER of combination therapy accounted for only 15.6% of this threshold, showing a prominent economic advantage. Despite regional economic disparities across China, combination therapy remained highly acceptable even at lower WTP levels. As China’s WTP threshold system continues to improve, the findings of this study can serve as a stable reference for healthcare reimbursement decisions and clinical medication selection in different regions.

Finerenone and empagliflozin exert complementary mechanisms of action: the former exerts anti-inflammatory and anti-fibrotic effects by blocking excessive mineralocorticoid receptor activation, while the latter achieves cardiorenal protection by alleviating renal hemodynamic load. The CONFIDENCE trial confirmed that their simultaneous initiation produced a synergistic renoprotective effect (1 + 1>2) with safety profiles comparable to monotherapy, no significant increase in adverse events such as hyperkalemia, and favorable clinical tolerability (Agarwal et al., 2022; Pitt et al., 2021).

Building on these clinical findings, this study further verified that the regimen offers both clinical benefits and cost-utility advantages. We recommend that healthcare security authorities further optimize reimbursement policies for this combination to expand clinical accessibility, reduce the patient burden of end-stage renal disease and dialysis, and improve the efficiency of overall health resource allocation.

### 4.4 Strengths

This study has four core strengths:

The model design reflects the natural progression of CKD, with a 1-year cycle and 30-year simulation horizon, consistent with the chronic progressive nature of the disease and more realistically reflecting patients’ lifetime disease burden.

The intervention was based on the CONFIDENCE trial—the first global RCT of simultaneous initiation of finerenone and empagliflozin—ensuring a high level of evidence. Parameter calibration using UACR reduction data from the Asian subgroup avoided bias from subjective assumptions.

The model strictly followed pharmacological mechanisms: only transitions related to kidney disease progression were calibrated, while unrelated pathways, such as disease regression and death, remained unchanged, thereby ensuring structural validity.

China-localized costs for medications, dialysis, and transplantation were used, and comprehensive sensitivity analyses confirmed model robustness, providing localized evidence for clinical and reimbursement decision-making in China.

### 4.5 Limitations

This study also has several limitations:

1. The model used constant transition probabilities over the 30-year horizon, without incorporating time-dependent effects, which may differ from real-world dynamics of disease progression.
2. Clinical efficacy parameters were mainly derived from the Asian subgroup of the CONFIDENCE trial. Although more relevant to Chinese patients, further validation using domestic real-world data is still needed.
3. Only direct medical costs were included in the cost analysis; indirect and intangible costs were excluded, potentially underestimating the comprehensive value of combination therapy.
4. Only empagliflozin monotherapy was used as the comparator, excluding other SGLT-2i or GLP-1RA-based combination regimens, underscoring the need to broaden the scope of future research.
5. The extrapolation of short-term UACR reduction from the CONFIDENCE trial to 30-year long-term disease progression probability in this study is based on the log-linear relationship between UACR change and CKD progression risk with strong theoretical support. However, uncertainty remains regarding whether short-term changes in UACR can fully capture the entire clinical benefit of long-term hard endpoints. Future validation of our findings is warranted when long-term hard endpoint follow-up data from the CONFIDENCE trial become available.
6. Utility values for NA and MAU states were conservatively estimated (NA = 0.936, MAU = 0.92) due to the lack of published Chinese utility data stratified by albuminuria grade. Sensitivity analyses confirmed that the uncertainty of this assumption did not alter the base-case conclusions. Future studies are needed to accumulate utility data for the Chinese population, stratified by albuminuria grade, to refine model estimates.
7. The Progression Coefficient (PC) was applied only to early albuminuria-related transitions. However, if combination therapy also delays progression to hemodialysis beyond its UACR-mediated effect, the base-case analysis may underestimate the full long-term value of the intervention. This should be revisited when long-term hard endpoint data from the CONFIDENCE trial become available.
8. Adverse events were simplified in the model, and the specific costs and disutilities associated with hyperkalemia monitoring and management were not separately incorporated. Future studies with more detailed safety data may refine this aspect of the model.

## 5 Conclusion

Based on the comprehensive analysis above, the simultaneous initiation regimen of finerenone plus empagliflozin derived from the CONFIDENCE study yields superior health outcomes and a significant cost-utility advantage compared with empagliflozin monotherapy for T2DM-CKD patients in a Markov model with a 1-year cycle and a 30-year simulation horizon. Sensitivity analyses confirm that the conclusions are highly robust. This regimen can provide high-quality evidence for standardized clinical treatment, rational medication use, the optimization of medical insurance payment policies, and the allocation of health resources for T2DM-CKD patients in China.

## Data Availability

The original contributions presented in the study are included in the article/supplementary material, further inquiries can be directed to the corresponding author.
